# Stable luminescent diphenylamine biphenylmethyl radicals with α-type D_0_ → D_1_ transition and antiferromagnetic properties†

**DOI:** 10.1039/d4sc08026b

**Published:** 2025-02-10

**Authors:** Shengxiang Gao, Chunxiao Wu, Ming Zhang, Feng Li

**Affiliations:** a State Key Laboratory of Supramolecular Structure and Materials, College of Chemistry, Jilin University Qianjin Avenue 2699 Changchun 130012 China lifeng01@jlu.edu.cn

## Abstract

Organic luminescent radicals, with their open-shell electronic structure, exhibit appealing optoelectronic properties and hold promise for a wide range of potential applications. Although a few series of luminescent radicals have been reported, most feature D_0_ → D_1_ transitions dominated by HOMOβ–SOMOβ (β-type transitions). In contrast, luminescent radicals that exhibit SOMOα–LUMOα properties (α-type transitions) are much rarer. Here, we designed and synthesized two stable novel diphenylamine biphenylmethyl (BTM) luminescent radicals, both characterized by α-type D_0_ → D_1_ transitions, and simultaneously maintained the D–A˙ structure for the first time. Density functional theory (DFT) calculations confirmed that fine-tuning the energy levels of frontier molecular orbitals can facilitate β-type to α-type D_0_ → D_1_ transition. Besides, in a 2(2Cl(m)PhN)-BTM crystal we for the first time observed strong antiferromagnetic interactions among luminescent radicals through SQUID measurements. This work offers design insights into luminescent radicals with α-type transition for future development.

## Abstract 2

Novel BTM luminescent radicals facilitate the luminescence transition from common β-type to rare α-type within a D–A˙ framework, enhancing the potential for developing new luminescent systems in the field of organic materials science.

## Graphical abstract

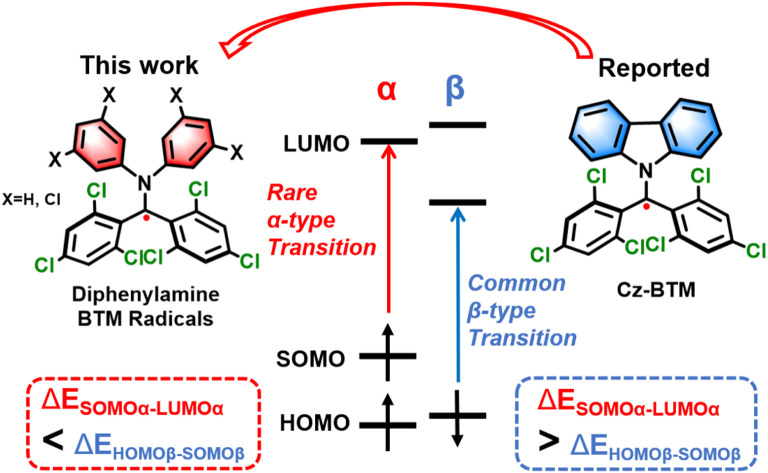

## Introduction

Organic luminescent radicals, characterized by their unique photo-electro-magnetic properties, have found widespread application in various research fields. Particularly noteworthy is their potential use in organic light-emitting diodes (OLEDs), where the spin-allowed doublet transition theoretically enables an internal quantum efficiency (IQE) approaching 100%.^1–7^ Most of the stable organic luminescent radicals reported to date fall within the category of chlorinated triphenylmethyl carbon radicals and their derivatives. These primarily include perchlorotriphenyl methyl radical derivatives (PTM),^8–10^ tris(2,4,6-trichlorophenyl)methyl radical derivatives (TTM),^11–14^ and pyridyl-containing triarylmethyl radicals (Py-BTM)^15–18^ and their derivatives. Besides, our group reported an (*N*-carbazolyl)bis(2,4,6-trichlorophenyl)-methyl radical (Cz-BTM)^19^ and a similar (*N*-pyrido[3,4-*b*]indolyl)bis(2,4,6-trichlorophenyl)methyl radical (PyID-BTM),^20^ in which the central carbon atoms of the radicals are directly bonded to nitrogen atoms. However, compared to the extensively studied TTM, PTM, and Py-BTM radical derivatives, Cz-BTM derivatives have received considerably less attention in the literature and with very limited reported derivatives.

**Scheme 1:**
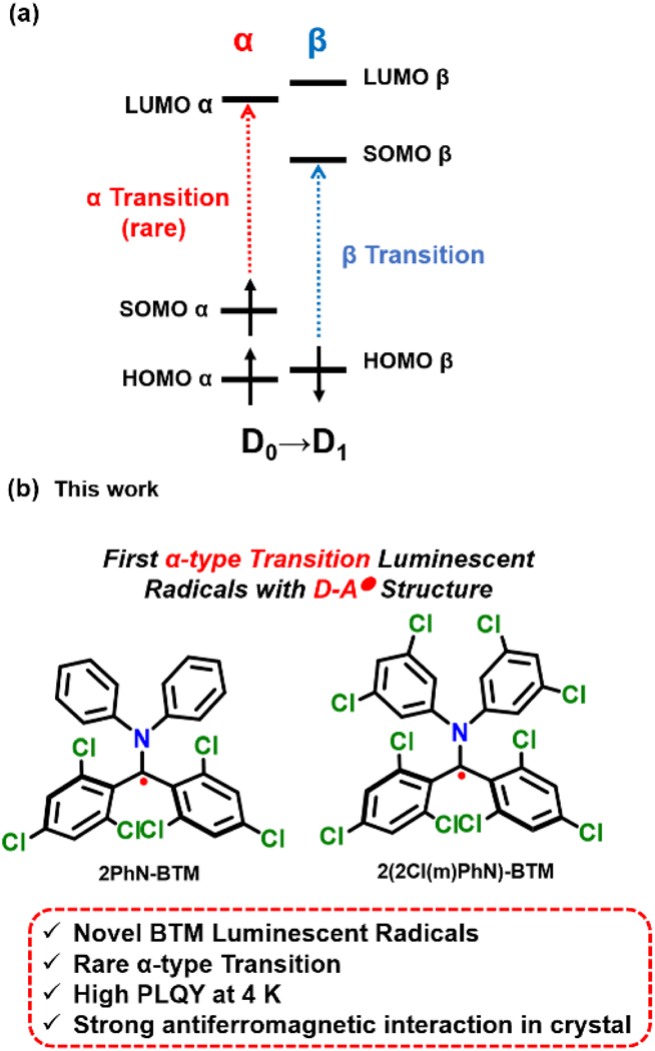
(a) Schematic representation of α and β transitions. (b) Structures of the stable luminescent diphenylamine biphenylmethyl radicals in this study.

In open-shell molecules, the presence of unpaired electrons causes all molecular orbitals to split into two sub-orbitals, α and β, each with opposite electron spins. Consequently, the transition to the first excited state (D_0_ → D_1_) of radicals can follow two possible excitation pathways: excitation of the unpaired electron from SOMOα to LUMOα, known as the α-type transition, or from HOMOβ to SOMOβ, known as the β-type transition (Scheme 1a).^21–23^ The luminescence process is the reverse of the corresponding excitation pathway. Currently, the most effective strategy for enhancing the stability and luminescence efficiency of luminescent radicals involves modifying the molecule with various electron donors to form donor–acceptor radicals (D–A˙). As a result, their first excited states typically exhibit significant charge transfer (CT) properties, with the electron primarily transitioning from HOMOβ (where the electron cloud is concentrated on the donor) to SOMOβ (where the electron cloud is concentrated on the radical), thus following the β-type transition pathway. Nearly all reported D–A type luminescent radicals exhibit β-type transitions. The luminescent radicals with α-type transitions reported are very rare, due to the highly complex design and synthesis which hinders further development and exploration.^24,25^ α-Type transition luminescent radicals have distinctive potential in researching new efficient luminescent radical systems and developing applications. For instance, in OLEDs some host materials with a shallower HOMO energy level can be used because of the shallower energy levels of SOMOα/LUMOα than those of HOMOβ/SOMOβ in radicals, which has the potential to expand the selection range of host materials. Developing α-type transition luminescent radicals with straightforward design and synthesis is also pivotal for broadening the diversity of radical luminescence, advancing the understanding of luminescence mechanisms, and gaining deeper insights into the photophysical processes involved.

In this work, we designed and synthesized two novel diphenylamine BTM luminescent radicals: (*N*-diphenylamine)bis(2,4,6-trichlorophenyl)methyl radical (2PhN-BTM) and (*N*-bis(3,5-dichlorophenyl)amine)bis(2,4,6-trichlorophenyl)methyl radical (2(2Cl(m)PhN)-BTM) (Scheme 1b). Theoretical calculations reveal that both 2PhN-BTM and 2(2Cl(m)PhN)-BTM exhibit α-type D_0_ → D_1_ transition, but maintain the radical moieties functioning as electron acceptors. Therefore, these novel diphenylamine BTM luminescent radicals are also the first α-type transition luminescent radicals with D–A˙ structure, which is distinct from previous α-type transition luminescent radicals where the radical moiety acts as an electron donor. This not only broadens the BTM luminescent radical system, but also confirms the simple design strategy by fine-tuning the energy levels of frontier molecular orbitals for developing luminescent radicals with α-type transition.

## Experimental

### Materials

All starting materials were obtained from commercial suppliers and used without further purification. The intermediate HBTM-Br and biphenylmethyl radicals were prepared by a previously reported method.^19,20^ Detailed synthesis procedures and characterization results are provided in the ESI.†

### Characterization

GC-MS mass spectra were recorded on a Thermo Fisher ITQ1100 mass spectrometer. The ^1^H and ^13^C nuclear magnetic resonance (NMR) spectra were recorded in a solution of CDCl_3_ on a Bruker AVANCEIII500 NMR spectrometer with TMS as the internal standard. Elemental analysis data were recorded on a Elementar Vario micro cube. EPR spectra were recorded on a Bruker ELEXSYSII E500 CW-EPR spectrometer in the ambient atmosphere. All photophysical measurements were performed in cyclohexane solution around 10^−5^ M at room temperature. A UV/Vis spectrophotometer (Shimadzu UV-2550) and spectrofluoro-photometer (Shimadzu RF-6000) were used to measure the absorption and fluorescence spectra in solution respectively. Single crystal X-ray diffraction data were collected on a Bruker Apex II CCD diffractometer at 293(2) K. The CV measurements were performed using an electrochemical analyzer (CHI660C, CH Instruments, USA) at the rate of 100 mV s^−1^. A glass carbon disk was used as the working electrode and a platinum wire acted as the counter electrode and Ag/AgCl acted as the reference electrode. Tetrabutylammonium hexafluorophosphate (TBAPF_6_) in anhydrous dichloromethane was used as the supporting electrolyte. The photoluminescence decay spectra measurements were carried out on Edinburgh Instruments spectrometer FLS980-S2S2-stm. Magnetic measurements were performed on a Quantum Design MPMS3-Zxin system with a temperature range of 1.9–300 K and an applied field of 0.5 T. The polycrystalline samples were prepared by a solvent evaporation method, ground and air dried. The amount used for testing was 0.03 mmol. The data were corrected for diamagnetism of the sample holder.

### Computational methods

DFT calculations were performed on a Gaussian09 series of programs using the (U)B3LYP function and 6-31G(d,p) basis, and using cyclohexane as the solvent in all calculations.^26^ Hole–electron analysis was performed on Multiwfn 3.8 program.^27,28^

## Results and discussion

### Synthesis and characterization

The synthesis of diphenylamine BTM radicals follows a similar pathway to that of the reported Cz/PyID-BTM radicals, utilizing a one-step reaction of diphenylamine derivatives with the intermediate HBTM-Br, as depicted in the ESI.† Consistent with Cz-BTM radical derivatives, both diphenylamine BTM radicals exhibit robust stability in the presence of oxygen, water, and light. They remain stable both as solids and in solution over extended periods under ambient conditions.

**Fig. 1:**
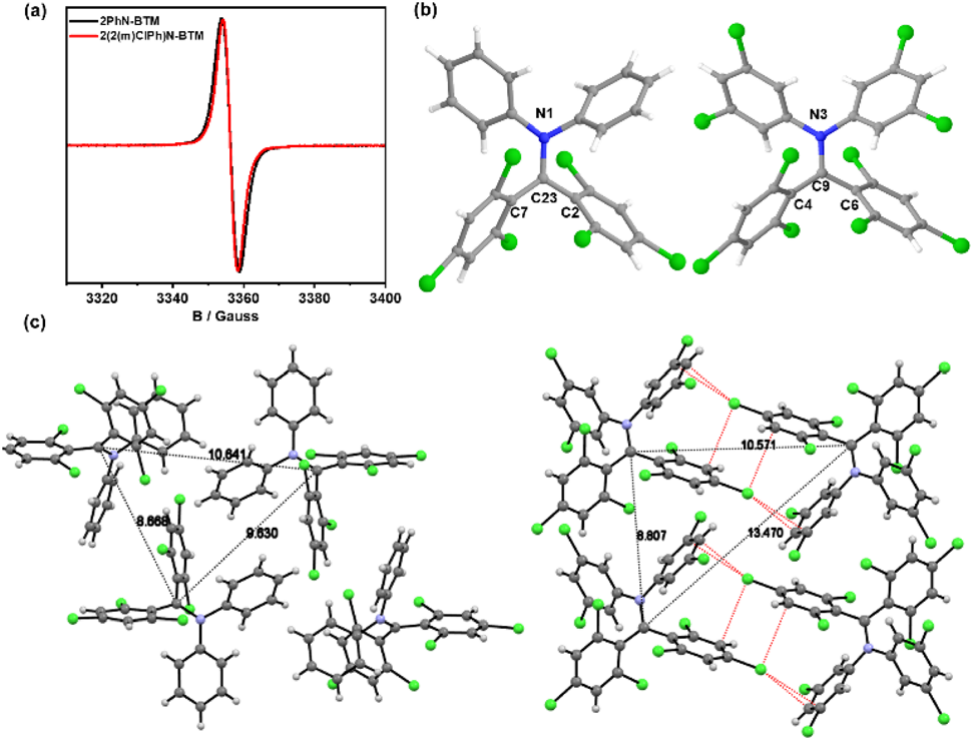
(a) EPR spectrum of 2PhN-BTM and 2(2Cl(m)PhN)-BTM in DCM solution (10^−3^ M) at room temperature. (b) Crystal structure of 2PhN-BTM and 2(2Cl(m)PhN)-BTM. (c) The crystal stacking structure of 2PhN-BTM and 2(2Cl(m)PhN)-BTM; black dashed lines: the distance between the central carbon atoms of adjacent radicals; red dashed lines: Cl⋯π halogen bonds between adjacent 2(2Cl(m)PhN)-BTM molecules.

EPR spectroscopy of 2PhN-BTM (Fig. 1a) confirmed the existence of an unpaired electron with *g* = 2.0038. The molecular structure in crystalline form was analyzed by single crystal X-ray diffraction (Table S1†). The single crystal of 2PhN-BTM was obtained through evaporation of cyclohexane/dichloromethane solution. The radical center atom, C23, is sterically shielded by four benzene rings and is coplanar with the adjacent N1, C2, and C7 atoms, confirming the sp^2^ hybridization of the radical center (Fig. 1b). However, significant decomposition was observed during the purification of 2PhN-BTM using silica gel column chromatography, resulting in a reduction in yield. This decomposition was partially mitigated by employing alumina column chromatography.

To enhance the stability of diphenylamine BTM radicals during purification, we explored incorporating chlorine atoms that are commonly used to stabilize triphenylmethyl radicals onto the benzene rings of diphenylamine. Despite the extensive steric hindrance provided by bis(2,4,6-trichlorophenyl)amine, this compound did not react with HBTM-Br to form the target radicals due to the increased activation energy caused by the substantial steric hindrance. Consequently, we modified the chlorine atoms to the meta-positions and successfully synthesized the target radical 2(2Cl(m)PhN)-BTM by reacting bis(3,5-dichlorophenyl)amine with HBTM-Br. This modification significantly improved the stability of 2(2Cl(m)PhN)-BTM during column chromatography, resulting in nearly complete avoidance of decomposition and a marked increase in yield. The purities of 2PhN-BTM (94%) and 2(2Cl(m)PhN)-BTM (95%) were determined *via* EPR measurements, employing 10^−4^ M TEMPO in toluene as an internal standard.

As shown in Fig. 1a, the EPR signal (*g* = 2.0038) of 2(2Cl(m)PhN)-BTM was similar to that of 2PhN-BTM. Due to the increased chlorine content reducing solubility in low-polarity solvents, single crystals were obtained by evaporation of tetrahydrofuran/acetonitrile solution. In the 2(2Cl(m)PhN)-BTM crystal, the radical center atom C9 is coplanar with N3, C4 and C6 atoms (Fig. 1b). The intermolecular distance between 2PhN-BTM molecules (measured from the central carbon atom) is 8.668 Å, 9.630 Å and 10.641 Å (Fig. 1c). For 2(2Cl(m)PhN)-BTM, these distances increase to 8.807 Å, 10.571 Å and 13.470 Å, indicating that the chlorine atoms on the benzene rings of diphenylamine provide steric hindrance effectively, protecting the radical center. In addition, 2(2Cl(m)PhN)-BTM presents multiple Cl⋯π halogen bonds between adjacent molecules in the crystal (Fig. 1c). This characteristic affects the magnetic properties of 2(2Cl(m)PhN)-BTM at low temperatures (see below). Moreover, the electron-withdrawing nature of the chlorine atoms enhances stability by lowering the energy levels of the frontier orbitals. Cyclic voltammetry (CV) measurements of 2PhN-BTM (Fig. S2†) suggest that SOMOα and SOMOβ energy levels are at −4.50 eV and −3.42 eV, respectively, whereas for 2(2Cl(m)PhN)-BTM, these values are at −4.86 eV and −3.64 eV. This reduction in SOMO energy levels confirms that the chlorine atoms effectively lower the energy levels of the frontier orbitals, thereby reducing reactivity and improving stability.

### Photophysical properties

**Fig. 2:**
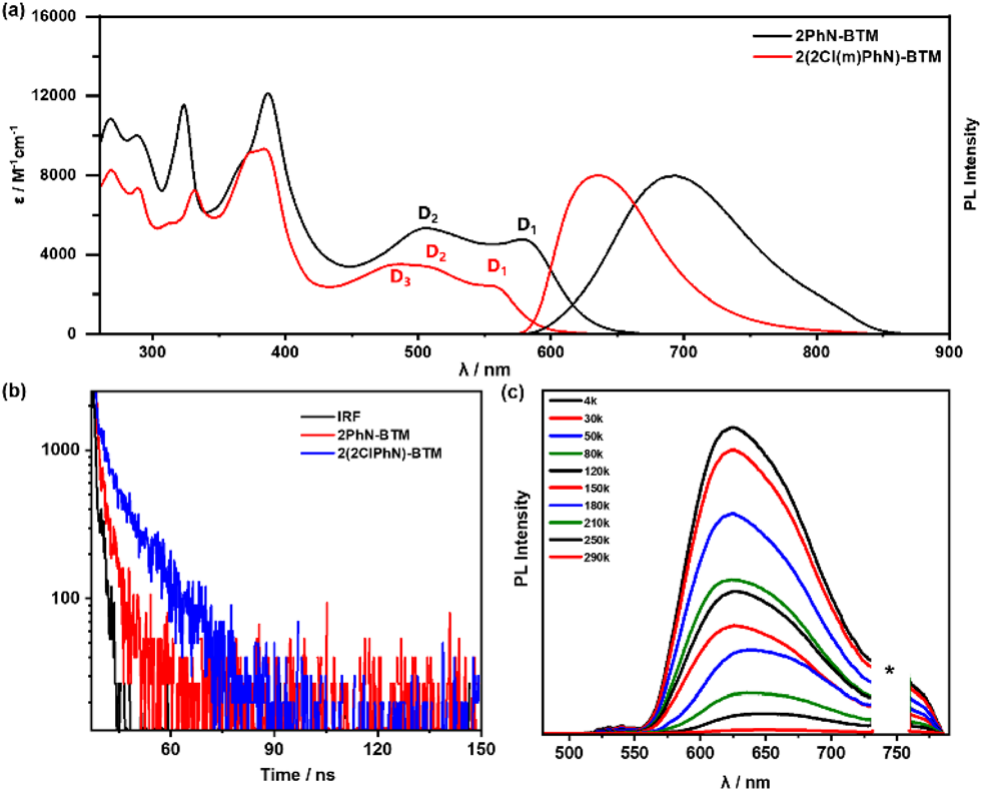
(a) Absorption and emission spectra of 2PhN-BTM and 2(2Cl(m)PhN)-BTM in cyclohexane solution (10^−5^ M) under excitation at 365 nm at room temperature. (b) Photoluminescence decay spectra of 2PhN-BTM and 2(2Cl(m)PhN)-BTM in cyclohexane solution (10^−5^ M) under excitation at 365 nm at room temperature. (c) Photoluminescence spectra of 2(2Cl(m)PhN)-BTM in cyclohexane solution at different temperatures under UV laser irradiation (365 nm) (the asterisk represents the harmonic peak of the light source).

Fig. 2a presents the UV/Vis absorption spectra of 2PhN-BTM and 2(2Cl(m)PhN)-BTM in cyclohexane. In the long-wavelength region (wavelengths > 450 nm), both compounds exhibit characteristic absorption peaks attributed primarily to transitions in the frontier orbitals. 2PhN-BTM exhibits two closely positioned absorption peaks at 506 nm and 579 nm. Time-dependent density functional theory (TD-DFT) calculations (UB3LYP, 6-31G(d,p)) assign these peaks to D_0_ → D_2_ and D_0_ → D_1_ excitations, respectively (Table S2†). The difference in peak intensities aligns with calculation predicted oscillator strengths. Notably, the D_0_ → D_1_ excitation process in 2PhN-BTM involves transitions predominantly from the SOMOα orbital (137α) to the LUMOα orbital (138α), contributing over 94% to the excitation, indicative of an α-type transition. This contrasts with traditional D–A˙ type luminescent radicals, where the first excited states typically involve HOMOβ–SOMOβ transitions (β-type transitions). The D_0_ → D_2_ excitation in 2PhN-BTM involves transitions from HOMOβ orbitals (136β) to SOMOβ orbitals (137β). Thus, the transition type of the first excited state is governed by the smaller energy gap between SOMOα–LUMOα and HOMOβ–SOMOβ. For 2(2Cl(m)PhN)-BTM, three primary absorption peaks in the long-wavelength region are observed at 483 nm, 506 nm, and 556 nm. This corresponds to the SOMOα–LUMO+1α transition (D_0_ → D_3_), the HOMOβ–SOMOβ transition (D_0_ → D_2_), and the SOMOα–LUMOα transition (D_0_ → D_1_), respectively. The absorption peak associated with the D_0_ → D_1_ transition of 2(2Cl(m)PhN)-BTM is slightly blue-shifted compared to 2PhN-BTM, and due to lower oscillator strength, its molar extinction coefficient is also reduced.

In cyclohexane under 550 nm excitation, 2PhN-BTM exhibits an emission band at 694 nm, comparable to that of the previously reported Cz-BTM. In contrast, 2(2Cl(m)PhN)-BTM shows a notable blue shift of 58 nm to 636 nm. The alignment of the main absorption peaks with the excitation spectra confirms the origin of the emission (Fig. S3†). However, the PLQY of 2PhN-BTM (0.14%) and 2(2Cl(m)PhN)-BTM (0.27%) are significantly lower than that of Cz-BTM (2.0%) under 550 nm excitation in cyclohexane at room temperature. This reduced PLQY is likely attributed to the lack of connectivity between the *N*-benzene rings of diphenylamine compared to Cz-BTM. The unobstructed rotation of the benzene rings in both radicals facilitates non-radiative decay pathways. The transient photoluminescence decay measurements at room temperature (Fig. 2b) reveal that 2PhN-BTM has a shorter lifetime (*τ*) of 3.14 ns compared to 2(2Cl(m)PhN)-BTM, which has a lifetime of 8.27 ns. The radiative rate constants (*k*_r_) and non-radiative rate constants (*k*_nr_) were calculated from PLQY and *τ* values (Table S3†). 2(2Cl(m)PhN)-BTM exhibits a significant reduction in *k*_nr_ compared to 2PhN-BTM, attributed to the steric hindrance of chlorine atoms that restricts the rotation of the benzene rings. Reorganization energy (*E*_R_) calculations for the excitation and de-excitation processes between D_0_ and D_1_ (Table S4†) indicate that 2(2Cl(m)PhN)-BTM has lower semi-reorganization energy than 2PhN-BTM, suggesting reduced non-radiative energy loss during the luminescence process. However, the *k*_r_ of 2(2Cl(m)PhN)-BTM is lower than that of 2PhN-BTM, primarily due to its lower oscillator strength for the first excited state. To investigate the impact of benzene ring rotation on the low PLQY, we measured the photoluminescence spectra of 2PhN-BTM and 2(2Cl(m)PhN)-BTM at reduced temperatures. As shown in Fig. S4,† the luminescence intensity of both compounds increased significantly at 80 K, with PLQYs rising to 1.5% and 8.6%, respectively. At 4 K, the PLQYs further increased to 2.6% and 16%, respectively (Fig. 2c and S5†). This substantial enhancement in PLQYs suggests that benzene ring rotation is effectively suppressed at low temperatures, thus reducing non-radiative transition rates.

We also evaluated the photo- and thermal stability of the compounds. Under 375 nm pulsed laser irradiation, both radicals presented much more enhanced photostability than the simplest luminescent radical TTM (Fig. S6†). 2PhN-BTM presented a fitted half-life (*t*_1/2_) of approximately 2.28 × 10^4^ s, which is almost identical to that of Cz-BTM (*t*_1/2_ = 2.13 × 10^4^ s), attributed to their similar molecular structure. Interestingly, despite 2(2Cl(m)PhN)-BTM showing enhanced stability during purification, 2PhN-BTM exhibited superior photostability, whereas 2(2Cl(m)PhN)-BTM displayed a shorter half-life (*t*_1/2_ = 4.33 × 10^3^ s), indicating reduced photostability. This decrease in photostability for 2(2Cl(m)PhN)-BTM is likely due to the additional chlorine atoms.^29,30^ The lower bond dissociation energy of the C–Cl bond (328 kJ mol^−1^) compared to the C–H bond (414 kJ mol^−1^) makes 2(2Cl(m)PhN)-BTM more prone to photochemical degradation under UV exposure, attributed to the cleavage of the C–Cl bond. Thermogravimetric analysis (TGA) revealed that 2(2Cl(m)PhN)-BTM exhibits slightly better thermal stability than 2PhN-BTM, likely related to the lower energy level of its frontier molecular orbitals (Fig. S7†).

### Theoretical calculations

**Fig. 3:**
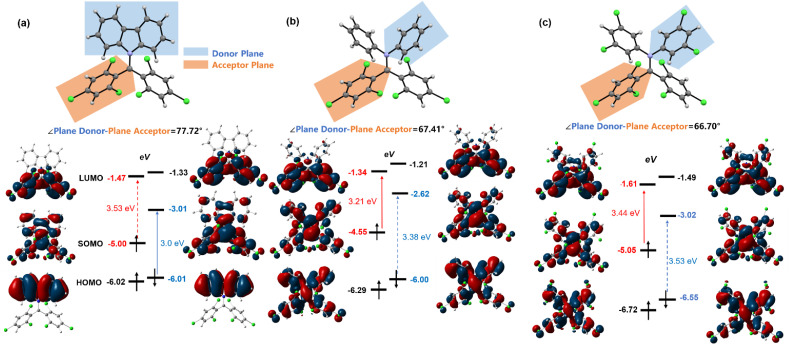
Donor/acceptor plane angles and frontier orbitals of Cz-BTM (a), 2PhN-BTM (b) and 2(2Cl(m)PhN)-BTM (c) according to DFT calculations at the ground state (UB3LYP, 6-31G(d,p)) (isovalue = 0.02).

We employed DFT calculations (UB3LYP, 6-31G(d,p)) to elucidate the energy levels and electron cloud distribution of frontier molecular orbitals in diphenylamine BTM radicals, aiming to understand the α-type transition mechanism (Fig. 3). For Cz-BTM, the β-type transition energy gap (Δ*E*_HOMOβ–SOMOβ_ = 3.0 eV) is smaller than the α-type transition energy gap (Δ*E*_SOMOα–LUMOα_ = 3.53 eV), making β-type transition the lowest energy excitation. For 2PhN-BTM, diphenylamine raises the energy levels of both SOMOs and LUMOs. The rise of SOMO energy levels is more pronounced, reducing Δ*E*_SOMOα–LUMOα_ to 3.21 eV. Simultaneously, the SOMOβ energy level increases to −2.62 eV while the HOMOβ remains nearly unchanged, which widens the β-type transition gap to 3.38 eV, exceeding the α-type transition gap. Consequently, 2PhN-BTM undergoes α-type transition. A similar pattern is observed in 2(2Cl(m)PhN)-BTM, where the additional chlorine atoms significantly lower the frontier orbital energies. Compared to Cz-BTM, the LUMO energy levels in 2(2Cl(m)PhN)-BTM are slightly reduced while the SOMO energy levels remain nearly unchanged. However, the HOMO energy levels decrease considerably. This results in a larger Δ*E*_HOMOβ–SOMOβ_ exceeding the Δ*E*_SOMOα–LUMOα_, which induces 2(2Cl(m)PhN)-BTM to undergo α-type transition. The Δ*E*_SOMOα–LUMOα_ and Δ*E*_HOMOβ–SOMOβ_ for 2(2Cl(m)PhN)-BTM are 3.44 eV and 3.53 eV, respectively; both are larger than those for 2PhN-BTM, consistent with the observed blue shift of its absorption and emission spectra. Furthermore, the plane of one trichlorobenzene at the radical moiety is defined as the acceptor plane and the plane of carbazole or one diphenylamine benzene ring as the donor plane (Fig. 3). For Cz-BTM, the HOMO electron clouds primarily localize on the electron-donating moiety due to the large dihedral angle (77.72°) between the acceptor and donor planes, which hinders electron cloud delocalization from the carbazole to the radical moiety. In contrast, for 2PhN-BTM and 2(2Cl(m)PhN)-BTM, the rotation of diphenylamine benzene rings reduces the angle between the acceptor and donor planes (67.41° for 2PhN-BTM and 66.70° for 2(2Cl(m)PhN)-BTM). As a result, the HOMO electron clouds become more delocalized across the entire molecule. Therefore, by selecting electron-donating groups with appropriate donating strengths and fine-tuning the molecular structure and orbital distribution, it is possible to modulate the α and β energy gaps and enable both α-type and β-type transitions of BTM radicals.

**Fig. 4:**
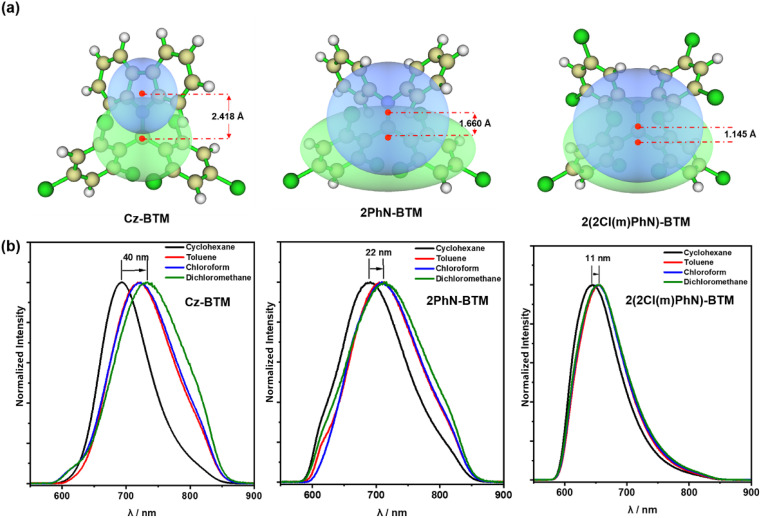
(a) Hole(blue)–electron(green) analysis of Cz-BTM, 2PhN-BTM and 2(2Cl(m)PhN)-BTM for D0 → D1 transition and distance between the hole center and electron center. (b) Photoluminescence spectra of Cz-BTM, 2PhN-BTM and 2(2Cl(m)PhN)-BTM in different solutions under 375 nm irradiation at room temperature.

Although 2PhN-BTM and 2(2Cl(m)PhN)-BTM exhibit α-type transition of D_0_ → D_1_, their charge transfer behavior remains similar to that of D–A˙ type luminescent radicals (for example, Cz-BTM). For Cz-BTM, upon excitation to the first excited state, the electron shifts from the carbazole moiety (HOMOβ orbital) to the entire molecule (SOMOβ orbital). For 2PhN-BTM, however, the charge transfer occurs from the SOMOα orbital, where the electron cloud is delocalized across the entire molecule, to the LUMOα orbital, where the electron cloud is primarily localized on the radical moiety. For 2(2Cl(m)PhN)-BTM, the electron-donating ability of the diphenylamine is significantly reduced, leading to a less pronounced charge transfer. We also analyzed the distribution of electrons (green) and holes (blue) during the D_0_ → D_1_ excitation process (Fig. 4a). For Cz-BTM, there is a substantial electron–hole separation with a distance of 2.418 Å. In contrast, 2PhN-BTM and 2(2Cl(m)PhN)-BTM exhibit more significant electron–hole overlap, indicating that their excitation processes are more characteristic of a localized excited (LE) state, with reduced electron–hole distances of 1.660 Å and 1.145 Å, respectively. The weakened CT characteristics are further corroborated by solvent effects (Fig. 4b). As solvent polarity increases from cyclohexane to dichloromethane, Cz-BTM exhibits a 40 nm red shift of its emission peak, indicating dominant CT characteristics, whereas 2PhN-BTM and 2(2Cl(m)PhN)-BTM exhibit smaller red shifts of only 22 nm and 11 nm, respectively, confirming their more pronounced LE characteristics.

### Magnetic properties

**Fig. 5:**
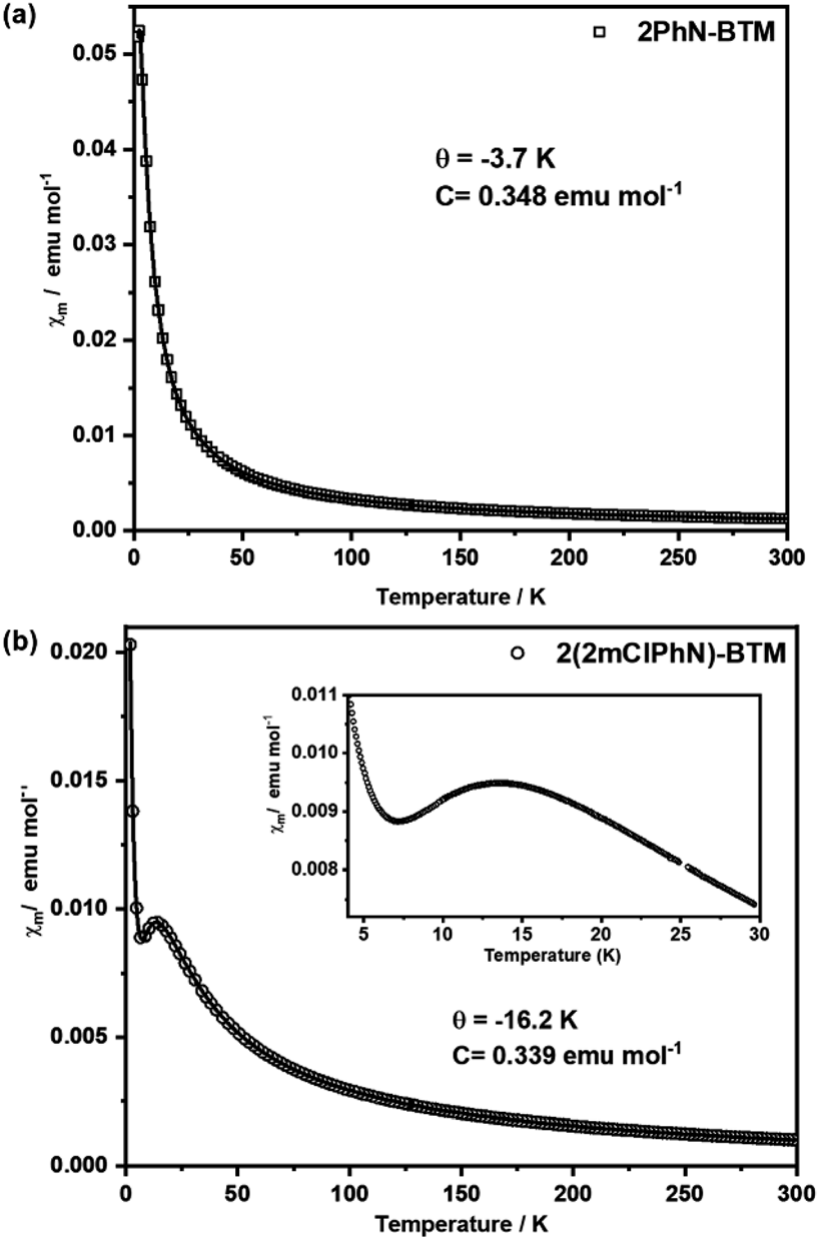
Molar magnetic susceptibility (*χ*_m_) *versus T* for (a) 2PhN-BTM and (b) 2(2Cl(m)PhN)-BTM at a temperature range of 1.9 K to 300 K.

The presence of unpaired electrons in luminescent radicals imparts unique paramagnetic properties. The magnetic behaviors of 2PhN-BTM and 2(2Cl(m)PhN)-BTM polycrystalline samples were investigated by using a superconducting quantum interference device (SQUID) magnetometer. Plots of mole magnetic susceptibility (*χ*_m_) *versus T* for both radicals are presented in Fig. 5. Consistent with some reported luminescent radicals, *χ*_m_ of 2PhN-BTM decreases rapidly with temperature increasing and follows the Curie–Weiss rule in the temperature range of 1.9 K to 300 K (Table S5†).^2,20,31–33^ The Curie constant *C* = 0.348 emu mol^−1^ is close to the expected value for *S* = 1/2 spin systems without spin interactions (0.375 emu mol^−1^), indicating dominant paramagnetic behavior in 2PhN-BTM. Additionally, the small and negative Weiss temperature *θ* of 2PhN-BTM (−3.7 K) suggests the presence of very week antiferromagnetic spin–spin interactions in the crystal.^31–33^ This is likely due to the distorted molecular structure of luminescent triaryl methyl radicals, which limits strong intermolecular interactions such as π⋯π stacking.

In contrast, 2(2Cl(m)PhN)-BTM exhibits a different magnetic behaviour. Upon cooling from 300 K, *χ*_m_ increases and reaches a maximum at around 13 K, followed by a decline as the temperature decreases further, indicating the occurrence of antiferromagnetic interactions.^34–38^ The distinct trend near 13 K highlights obvious antiferromagnetic interactions in the 2(2Cl(m)PhN)-BTM crystal at low temperature. When the temperature is below 7 K, *χ*_m_ increases sharply because of uncoupled radicals at crystal lattice defect sites.^35,38,39^ The *χ*_m_^−1^*versus T* plot for 2(2Cl(m)PhN)-BTM has good linearity between 22 K and 300 K and adheres to the Curie–Weiss rule (Fig. S8†).^34,35,38^ Both the smaller *C* = 0.339 emu mol^−1^ and more negative *θ* = −16.2 K compared to 2PhN-BTM indicate stronger antiferromagnetic interactions in 2(2Cl(m)PhN)-BTM. Furthermore, temperature-dependent magnetic susceptibility (*χ*_m_*T*) *versus T* for 2PhN-BTM and 2(2Cl(m)PhN)-BTM also reveals similar results, in which 2PhN-BTM has a larger *χ*_m_*T* value at room temperature than 2(2Cl(m)PhN)-BTM (Fig. S9†). Despite the larger intermolecular central carbon atom distances of 2(2Cl(m)PhN)-BTM as shown in Fig. 1c, Cl⋯π halogen bonds between adjacent molecules enhance the intermolecular spin–spin interactions, contributing to the stronger antiferromagnetic behavior observed. Notably, this result marks the first observation of strong antiferromagnetic interaction characteristic of stable luminescent radicals, providing valuable insights into the interplay between molecular structure and magnetic behavior of organic luminescent radicals.

## Conclusions

In summary, we successfully designed and synthesized stable diphenylamine BTM luminescent radicals, 2PhN-BTM and 2(2Cl(m)PhN)-BTM, which exhibit the rare α-type D_0_ → D_1_ excitation while first maintaining D–A˙ structure. Theoretical calculations suggest that the α-type transition is achieved by fine-tuning the energy levels of frontier orbitals and adjusting the relative values of the α and β energy gaps. This suggests that the conversion between α-type and β-type transition in luminescent radicals can be strategically controlled through molecular design. 2(2Cl(m)PhN)-BTM demonstrated a significant increase in PLQY to 16% at 4 K. SQUID measurements revealed distinct magnetic properties between 2PhN-BTM and 2(2Cl(m)PhN)-BTM, which is attributed to the differences in their crystal structures. And, 2(2Cl(m)PhN)-BTM is found to be the first luminescent radical that exhibits strong antiferromagnetic interactions in the crystal. Future research will focus on enhancing the room temperature PLQYs of diphenylamine BTM radicals while preserving the α-type transition properties, and developing distinctive photophysical properties and application of α-type transition properties. This work presented a novel approach to designing α-type transition luminescent radicals.

## Data availability

Data for this article, including synthetic procedures, details and characterization data, crystallographic data and calculation results, are available at DOI: https://doi.org/10.1039/d4sc08026b. The data supporting this article have been included as part of the ESI.† Crystallographic data for 2PhN-BTM and 2(2Cl(m)PhN)-BTM have been deposited at the Cambridge Crystallographic Data Centre (CCDC) under CCDC numbers: 2384400 and 2384389,† respectively.

## Author contributions

Shengxiang Gao initiated the study, completed the experiments and analysis, and wrote the draft of the manuscript. Feng Li and Ming Zhang conceived and supervised the study, and reviewed and revised the manuscript. Chunxiao Wu provided experimental suggestions and participated in the data analysis.

## Conflicts of interest

The authors declare no competing financial interests.

## Supplementary Material

SC-OLF-D4SC08026B-s001

SC-OLF-D4SC08026B-s002
