# Supplementary material for: Stable luminescent diphenylamine biphenylmethyl radicals with α-type D0 → D1 transition and antiferromagnetic properties
Source: Chem Sci. 2025 Feb 10;16(11):4668–75. doi: 10.1039/d4sc08026b (PMC11815340; doi:10.1039/d4sc08026b)
Supplement: SC-016-D4SC08026B-s001 [file SC-016-D4SC08026B-s001.pdf]

Supplementary Information (SI) for Chemical Science.
This journal is © The Royal Society of Chemistry 2025

Stable Luminescent Diphenylamine Biphenylmethyl Radicals with α-Type $D_0 \rightarrow D_1$ Transition and Antiferromagnetic Properties

Shengxiang Gao, Chunxiao Wu, Ming Zhang, and Feng Li*

State Key Laboratory of Supramolecular Structure and Materials, College of Chemistry, Jilin University, Changchun 130012

* E-mail: lifeng01@jlu.edu.cn

Content

S1 Synthetic procedures, details and characterization data.

S2 Crystallographic data.

S3 Cyclic voltammetry (CV) measurements.

S4 DFT / TD-DFT theoretical calculations.

S5 Photophysical measurements.

S6 Stability.

S7 Reorganization energy (E_R) calculation.

S8 SQUID measurements.

S1 Synthetic procedures, details and characterization data.

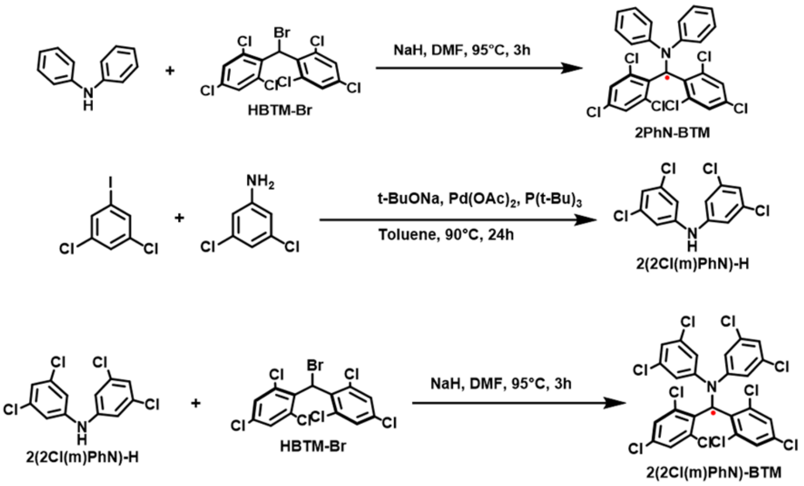

Scheme S1. Synthesis of 2PhN-BTM and 2(2Cl(m)PhN)-BTM.

S1.1 Synthesis 2PhN-BTM

Under argon atmosphere, sodium hydride (60% in oil, 0.06 g, 1.6 mmol) was dispersed in 2ml anhydrous cyclohexane and was stirred for 1min. Then 20 ml anhydrous DMF was added. Diphenylamine (0.27 g, 1.6 mmol) dissolved in anhydrous DMF (5 ml) was added dropwise and stirred for 1h. Then HBTM-Br (0.5 g, 1.1 mmol) was added. The mixture was heated to 95°C with stirred for 3 h. After cooling to room temperature 50ml saturated ammonium chloride solution was added. The precipitate was collected by suction filtration and purified by flash column chromatography (Neutral aluminum trioxide gel, petroleum ether/dichloromethane = 10:1). Atropurpureus solid 2PhN-BTM was obtained (87 mg, 15%). GC-MS (m/z): [M] calculated for $C_{25}H_{14}Cl_6N$, 539.92, found, 539.88. Elem. Anal. Calcd for C, 55.49; H, 2.61; N, 2.59; found, C, 55.66; H, 2.82; N, 2.33.

S1.2 Synthesis 2(2Cl(m)PhN)-BTM

Synthesis of compound 2(2Cl(m)PhN)-H. Under argon atmosphere 1,3-dichloro-5-iodobenzene (2.73 g, 10mmol), 3,5-dichloroaniline (1.62 g, 10 mmol), sodium tert-butoxide (0.96 g, 10 mmol) were dissolved in 20 ml toluene. The mixture was replaced with nitrogen for three times. Then palladium acetate (0.09 g, 0.4 mmol) and tri-tert-butyl phosphine (2 ml, 10w/v% in toluene). The mixture was heated to 90°C with stirred for 24 h. After cooling to room temperature, the solvents were removed by rotary evaporation. The residue was purified by column chromatography (silica gel, petroleum ether/ethyl acetate = 10:1). White solid 2(2Cl(m)PhN)-H was obtained (2.12 g, 69%). GC-MS (m/z): [M]+ calcd for $C_{12}H_7Cl_4N$, 307.00; found, 307.09. ^{1}H NMR (500 MHz, $CDCl_3$) δ 6.98 (t, J = 1.7 Hz, 2H), 6.92 (d, J = 1.7 Hz, 4H), 5.77 (s, 1H). ^{13}C NMR (500 MHz, $CDCl_3$) δ 144.08, 135.90, 122.19, 116.53.

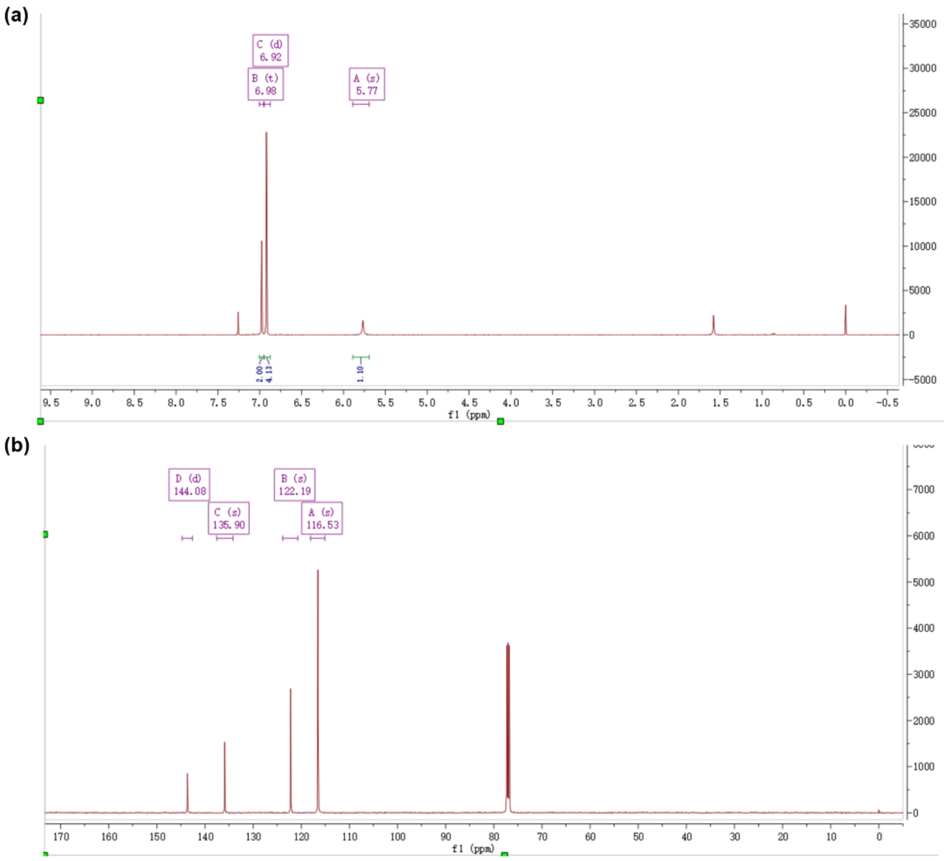

Figure S1. (a) ^{1}H NMR Spectrum and (b) ^{13}C NMR Spectrum (500 MHz, CDCl3) of 2(2Cl(m)PhN)-H.

Synthesis of 2(2Cl(m)PhN)-BTM. 2(2Cl(m)PhN)-H was treated as diphenylamine above: deep red solid 2(2Cl(m)PhN)-BTM was obtained (356 mg, 48%). GC-MS (m/z): [M] calculated for $C_{25}H_{10}Cl_{10}N$, 678.86, found, 678.32. Elem. Elem. Anal. Calcd for C, 44.23; H, 1.48; N, 2.06; found, C, 44.31; H, 1.57; N, 1.99. The significantly improved yield of 2(2Cl(m)PhN)-BTM compared to the Cz-BTM series of radicals in previously reported can be attributed to the flexible diphenylamine structure is more reactive, as opposed to the carbazole derivatives that possess a rigid structure. Additionally, 2(2Cl(m)PhN)-BTM also maintains good stability during the isolation process.

S2 Crystallographic data.

Table S1. Crystallographic data of 2PhN-BTM and 2(2Cl(m)PhN)-BTM.

Identification code	2PhN-BTM	2(2Cl(m)PhN)-BTM
CCDC	2384400	2384389
Empirical formula	$C_{25}H_{14}Cl_{6}N$	$C_{25}H_{10}Cl_{10}N$
Fw / g mol^{-1}	541.07	678.84
Temperature/K	125.0	291(2)
Crystal system	monoclinic	triclinic
Space group	$P2_1/c$	P-1
a/Å	16.4717(16)	8.3050(15)
b/Å	8.3566(9)	8.8068(17)

c/Å	17.0028(18)	19.111(4)
α/°	90	97.232(7)
β/°	103.423(4)	96.934(7)
γ/°	90	95.160(7)
Volume/Å^3	2276.5(4)	1368.7(5)
Z	4	2
ρ_{calc}g/cm^3	1.579	1.647
μ/mm^{-1}	0.770	1.037
F(000)	1092.0	674.0
Radiation	MoKα (λ = 0.71073)	MoKα (λ = 0.71073)
Reflections collected	64972	44359
GoF	1.056	1.137

S3 Cyclic voltammetry (CV) measurements.

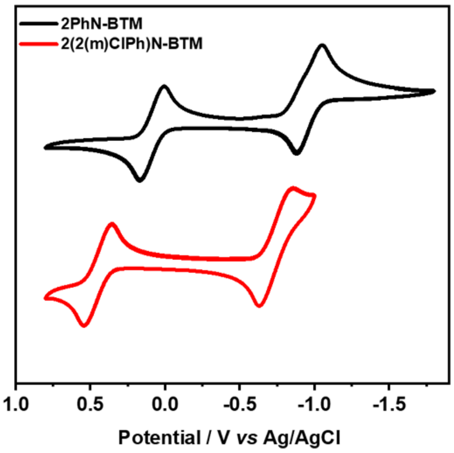

Figure S2. Cyclic voltammogram (CV) curves of 2PhN-BTM and 2(2Cl(m)PhN)-BTM in DCM solution with 100 mV/s scanning rate at room temperature.

S4 DFT / TD-DFT theoretical calculations.

Table S2. Summary of TD-DFT calculated excitation energies, oscillator strength and contributions of major orbitals transitions of $D_0 \rightarrow D_n$ excitation of 2PhN-BTM and 2(2Cl(m)PhN)-BTM.

	Transition	Wavelength (nm)	Energy (eV)	Osc.Strength (*f*)	Major Contribution (Proportion)
2PhN-BTM	$D_0 \rightarrow D_1$	533.16	2.3255	0.0827	137A ->138A (94%)
	$D_0 \rightarrow D_2$	503.90	2.4605	0.1293	136B ->137B (95%)
2(2Cl(m)PhN)-BTM	$D_0 \rightarrow D_1$	496.15	2.4989	0.0590	169A ->170A (90%)
	$D_0 \rightarrow D_2$	477.00	2.5992	0.1111	168B ->169B (92%)
	$D_0 \rightarrow D_3$	455.12	2.7242	0.0556	169A ->171A (91%)

Cartesian coordinates of all the optimized geometries by DFT calculation.

2PhN-BTM.

Center Number	Atomic Number	Atomic Type	Coordinates (Angstroms) X	Y	Z
1	6	0	0.000123	0.051197	0.000107
2	6	0	1.281479	-0.662310	-0.120876
3	6	0	2.238883	-0.371294	-1.129409
4	6	0	3.463394	-1.020721	-1.235592
5	1	0	4.148939	-0.763001	-2.032572
6	6	0	3.772337	-2.027190	-0.325893
7	6	0	2.876877	-2.381543	0.676520
8	1	0	3.119621	-3.157204	1.391202
9	6	0	1.668360	-1.697951	0.768006
10	6	0	-1.281286	-0.662383	0.121062
11	6	0	-1.668251	-1.697743	-0.768081
12	6	0	-2.876854	-2.381243	-0.676718
13	1	0	-3.119697	-3.156726	-1.391560
14	6	0	-3.772198	-2.027076	0.325848
15	6	0	-3.463143	-1.020905	1.235825
16	1	0	-4.148636	-0.763358	2.032906
17	6	0	-2.238573	-0.371544	1.129755
18	17	0	1.884327	0.788759	-2.398960
19	17	0	5.308530	-2.862027	-0.448859
20	17	0	0.642315	-2.146848	2.124412
21	17	0	-0.642236	-2.146515	-2.124505
22	17	0	-5.308492	-2.861802	0.448656
23	17	0	-1.883846	0.788113	2.399567
24	7	0	-0.000047	1.444340	0.000055
25	6	0	-1.051190	2.172165	-0.641529
26	6	0	-1.557887	1.743709	-1.877346
27	6	0	-1.589958	3.317343	-0.034332
28	6	0	-2.594716	2.447450	-2.487432
29	1	0	-1.128313	0.874347	-2.360595
30	6	0	-2.616443	4.020387	-0.659631
31	1	0	-1.208060	3.645452	0.925352
32	6	0	-3.128342	3.588337	-1.885711
33	1	0	-2.975815	2.106924	-3.445560
34	1	0	-3.026268	4.903165	-0.178064
35	6	0	1.050930	2.172571	0.641450
36	6	0	1.588941	3.318186	0.034386
37	6	0	1.558264	1.744103	1.877021
38	6	0	2.615253	4.021609	0.659535

39	1	0	1.206617	3.646326	-0.925119
40	6	0	2.594926	2.448239	2.486948
41	1	0	1.129360	0.874410	2.360268
42	6	0	3.127770	3.589554	1.885360
43	1	0	3.024467	4.904695	0.178016
44	1	0	2.976493	2.107667	3.444874
45	1	0	-3.932300	4.136565	-2.366883
46	1	0	3.931568	4.138101	2.366436

2(2Cl(m)PhN)-BTM.

Center Number	Atomic Number	Atomic Type	Coordinates (Angstroms) X	Y	Z
1	6	0	-0.000022	-0.654911	-0.000029
2	6	0	-1.221608	-1.364974	0.413055
3	6	0	-1.912556	-1.081451	1.620759
4	6	0	-3.078250	-1.733187	2.006179
5	1	0	-3.556587	-1.483666	2.944505
6	6	0	-3.595325	-2.729663	1.183620
7	6	0	-2.963599	-3.072054	-0.006812
8	1	0	-3.372000	-3.838177	-0.653026
9	6	0	-1.808265	-2.389228	-0.373486
10	6	0	1.221564	-1.364981	-0.413110
11	6	0	1.808186	-2.389259	0.373421
12	6	0	2.963525	-3.072087	0.006762
13	1	0	3.371899	-3.838232	0.652967
14	6	0	3.595288	-2.729668	-1.183642
15	6	0	3.078247	-1.733166	-2.006190
16	1	0	3.556615	-1.483622	-2.944494
17	6	0	1.912547	-1.081430	-1.620786
18	17	0	-1.268603	0.071768	2.777276
19	17	0	-5.058695	-3.565077	1.656329
20	17	0	-1.129404	-2.818790	-1.935934
21	17	0	1.129261	-2.818862	1.935829
22	17	0	5.058665	-3.565081	-1.656330
23	17	0	1.268641	0.071835	-2.777284
24	7	0	-0.000005	0.743643	-0.000006
25	6	0	1.166786	1.471073	0.381398
26	6	0	1.936633	1.042822	1.471933
27	6	0	1.546891	2.608763	-0.346177
28	6	0	3.081914	1.757901	1.808119
29	1	0	1.635603	0.182655	2.054694

30	6	0	2.688512	3.303085	0.037073
31	1	0	0.968936	2.936079	-1.200195
32	6	0	3.481266	2.896098	1.109931
33	6	0	-1.166768	1.471125	-0.381385
34	6	0	-1.546808	2.608832	0.346198
35	6	0	-1.936660	1.042911	-1.471904
36	6	0	-2.688404	3.303207	-0.037029
37	1	0	-0.968822	2.936123	1.200204
38	6	0	-3.081914	1.758042	-1.808066
39	1	0	-1.635682	0.182733	-2.054676
40	6	0	-3.481201	2.896258	-1.109870
41	1	0	4.371279	3.444026	1.390655
42	1	0	-4.371195	3.444226	-1.390574
43	17	0	-3.163590	4.727461	0.874067
44	17	0	-4.045721	1.223867	-3.175999
45	17	0	3.163783	4.727317	-0.874012
46	17	0	4.045669	1.223681	3.176071

Cz-BTM.

Center Number	Atomic Number	Atomic Type	Coordinates (Angstroms) X	Y	Z
1	6	0	-0.000002	0.069500	-0.000067
2	6	0	-1.285352	0.771525	-0.092009
3	6	0	-2.270856	0.436485	-1.060013
4	6	0	-1.645395	1.832024	0.779642
5	6	0	-3.501709	1.076932	-1.144898
6	6	0	-2.863106	2.501046	0.707294
7	6	0	-3.787087	2.109195	-0.255755
8	1	0	-4.212533	0.790201	-1.909143
9	1	0	-3.090154	3.296482	1.405195
10	6	0	1.285277	0.771643	0.091933
11	6	0	2.270789	0.436610	1.059927
12	6	0	1.645241	1.832242	-0.779627
13	6	0	3.501584	1.077160	1.144893
14	6	0	2.862890	2.501371	-0.707195
15	6	0	3.786885	2.109526	0.255845
16	1	0	4.212421	0.790422	1.909122
17	1	0	3.089883	3.296883	-1.405027
18	6	0	-0.879714	-2.157768	0.728310
19	6	0	0.879922	-2.157724	-0.728343
20	6	0	-1.884089	-1.815423	1.635829

21	6	0	-0.558291	-3.508748	0.462549
22	6	0	1.884276	-1.815335	-1.635869
23	6	0	0.558612	-3.508720	-0.462526
24	1	0	-2.114136	-0.784553	1.871459
25	6	0	-1.275044	-4.532309	1.088505
26	6	0	2.589135	-2.851959	-2.246971
27	1	0	2.114244	-0.784457	-1.871538
28	6	0	1.275453	-4.532248	-1.088435
29	6	0	-2.295866	-4.197133	1.974756
30	1	0	-1.033211	-5.572460	0.891450
31	6	0	2.296252	-4.197025	-1.974695
32	1	0	3.377402	-2.607345	-2.952314
33	1	0	1.033710	-5.572411	-0.891333
34	1	0	-2.863155	-4.980742	2.467169
35	1	0	2.863608	-4.980608	-2.467072
36	7	0	0.000068	-1.323585	-0.000033
37	17	0	-1.945786	-0.766390	-2.293385
38	17	0	-0.577842	2.325026	2.085003
39	17	0	-5.331354	2.928941	-0.352839
40	17	0	0.577684	2.325247	-2.084984
41	17	0	1.945808	-0.766438	2.293154
42	17	0	5.331075	2.929405	0.353029
43	6	0	-2.588860	-2.852080	2.246977
44	1	0	-3.377142	-2.607501	2.952315

S5 Photophysical measurements.

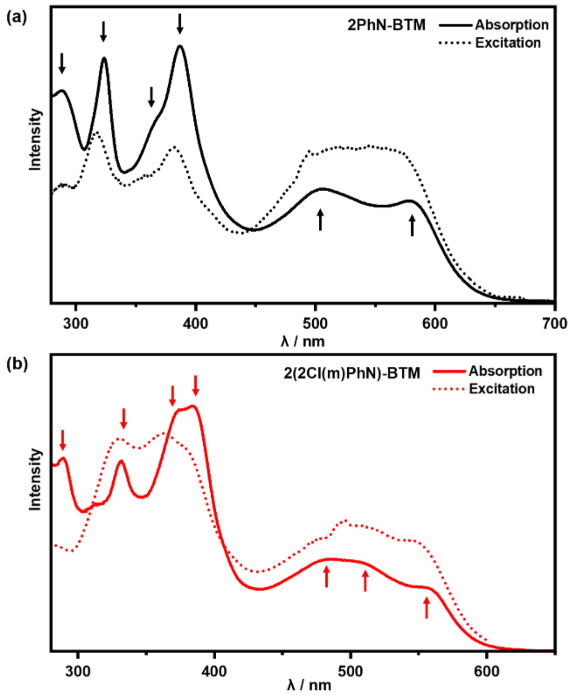

Figure S3. Absorption and excitation spectra of (a) 2PhN-BTM (luminescence set at 694 nm) and (b) 2(2Cl(m)PhN)-BTM (luminescence set at 636 nm) in cyclohexane, with arrows indicating the alignment of the main absorption peaks and excitation spectra.

Table S3. Photophysical parameters of 2PhN-BTM and 2(2Cl(m)PhN)-BTM in cyclohexane.

Radicals	λ_{pL}(nm)[a]	$ø_f$ (%)[a]	$ø_f$ (%)[b]	$ø_f$ (%)[c]	τ (ns)[a]	k_r (*10^5 s^{-1})[a]	k_{nr} (*10^8 s^{-1})[a]
2PhN-BTM	694	0.14%	1.5%	2.6%	3.14	4.46	3.18
2(2Cl(m)PhN)-BTM	636	0.27%	8.6%	16%	8.27	3.26	1.21

[a] At room temperature. [b] At 80 K. [c] At 4 K.

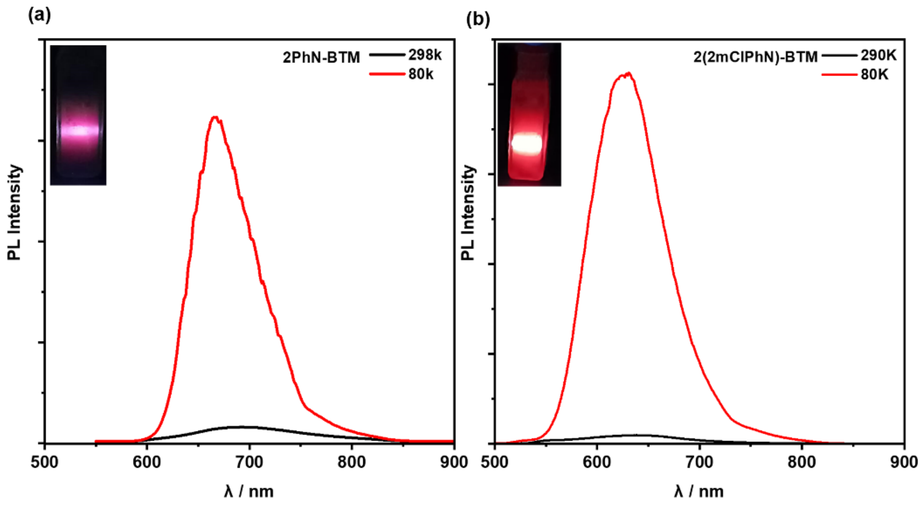

Figure S4. Photoluminescence spectra of 2PhN-BTM(a) and 2(2Cl(m)PhN)-BTM(b) in cyclohexane solution at 298K and 80K, respectively (the inset shows the photographs of 2PhN-BTM (a) and 2(2Cl(m)PhN)-BTM (b) under UV laser irradiation (365 nm).

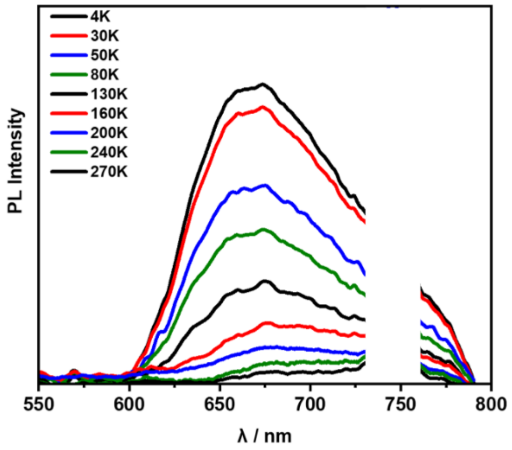

Figure S5. Photoluminescence spectra of 2PhN-BTM in cyclohexane solution at different temperature under UV laser irradiation (365 nm) (the asterisk represents the harmonic peak of the light source).

S6 Stability.

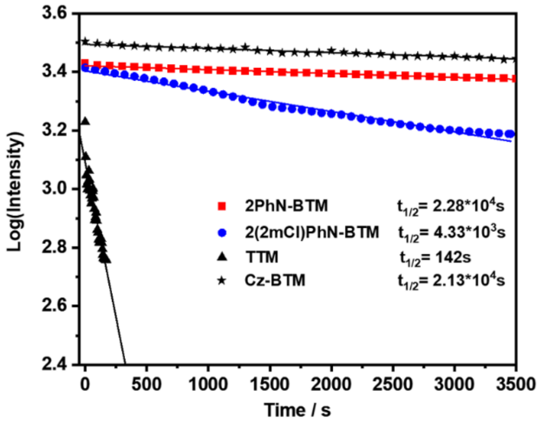

Figure S6. Time dependence of the emission intensities of 2PhN-BTM, 2(2Cl(m)PhN)-BTM, TTM and Cz-BTM in cyclohexane under 375 nm laser radiation.

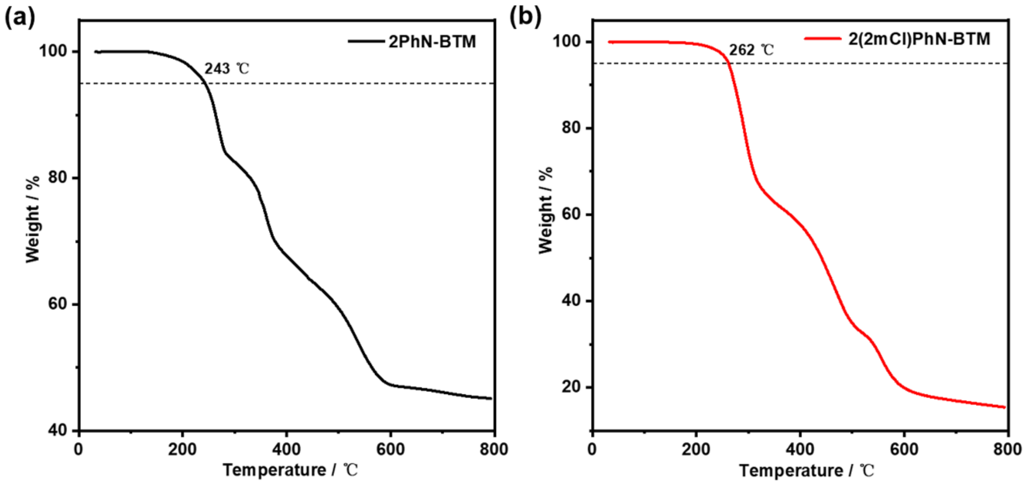

Figure S7. TGA curve of (a) 2PhN-BTM and (b) 2(2Cl(m)PhN)-BTM under nitrogen flow (the dashed lines indicate remaining 95% mass).

S7 Reorganization energy (E_R) calculation.

The calculation method for the reorganization energy between the D_0 state and the D_1 excited state of the molecule is shown in Scheme S2. The entire process from D_0 state to the first excited state through excitation, and then back to the ground state through de-excitation process, is divided into the following four stages (Scheme S2)：

I：Molecule in D_0 state stable configuration (energy is E_1) is excited to the D_1 state (energy is E_2) according to the Frank-Condon principle, and the configuration remains unchanged.

II：The excited molecule relaxes to the stable configuration of the D_1 state (energy is E_3)，semi-reorganization energy upon excitation (λ1) is the difference

between E_2 and E_3 ($\lambda 1 = E_2 - E_3$).

III: The molecular configuration in the first excited state remains unchanged and transitions back to the ground state, with energy of E4。

IV：The molecule in ground state relaxes to the stable configuration, and the semi-reorganizationenergy upon de-excitation (λ2) is the difference between E4 and E1 (λ1=E4-E1).

The total relaxation energy (E_R) of the entire process is the sum of λ1 and λ2. All calculations are using DFT method and results are as shown in Table S4.

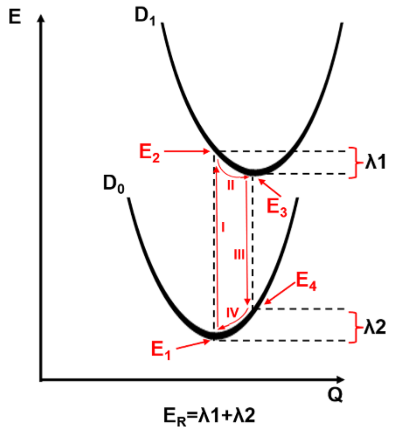

Scheme S2. Schematic diagram of reorganization energy calculation.

Table S4. The calculated semi-reorganization energy λ1, λ2 and total relaxation energy (E_R) of radicals.

E(eV)	λ1	λ2	E_R (λ1+λ2)
2PhN-BTM	0.3769	0.5374	0.9143
2(2Cl(m)PhN)-BTM	0.3410	0.5237	0.8647

S8 SQUID measurements.

The expression of Curie-Weiss rule is as:

$$\chi_m = \frac{C}{T - \theta}$$

where the χ_m is molar magnetic susceptibility, C is Curie constant, T is temperature and θ is Weiss temperature.

Table S5. The fitting results of χ_m versus T for 2PhN-BTM and 2(2Cl(m)PhN)-BTM by using Curie-Weiss rule.

Radicals	T range	C	θ	R^2
2PhN-BTM	1.9 K-300 K	0.34757	-3.74133	0.99676
2(2Cl(m)PhN)-BTM	22 K-300 K	0.33920	-16.18512	0.99943

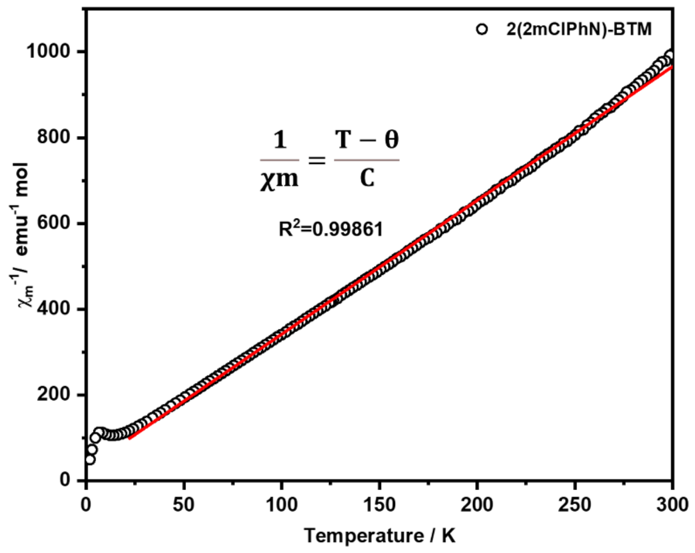

Figure S8. χ_m^{-1} versus T for 2(2Cl(m)PhN)-BTM at the temperature of 22 K to 300 K by fitting the Curie-Weiss rule.

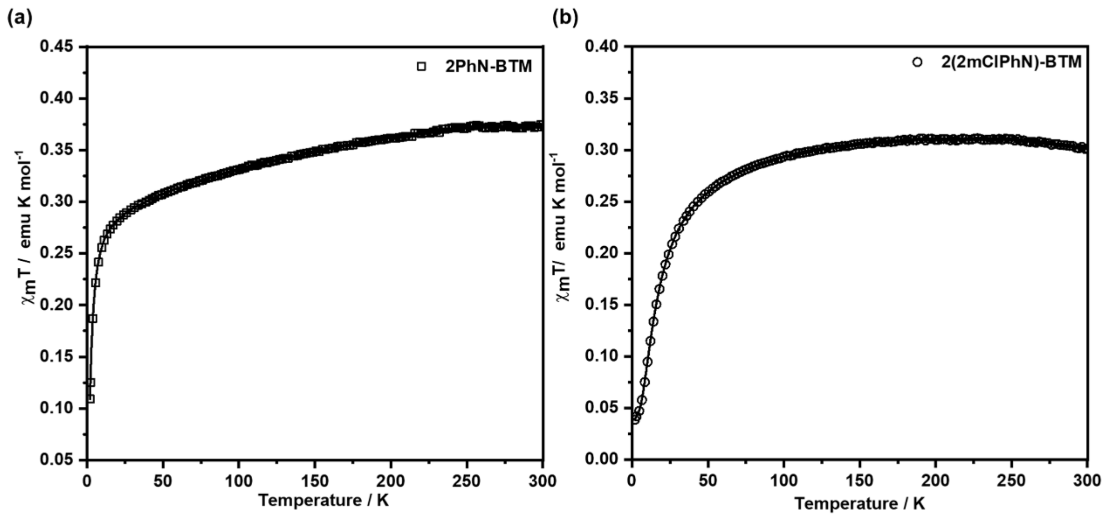

Figure S9. χ_mT versus T for for (a) 2PhN-BTM and (b) 2(2Cl(m)PhN)-BTM at T range of 1.9 K to 300 K.
